# Statistics of Age

**Published:** 1879-12

**Authors:** 


### STATISTICS OF AGE.

From some statistics collected in a Berlin paper, as to the duration of life, and especially as regards the proportion of the population which reaches an extreme old age, it appears that, for its population, Greece possesses the greatest number of very old people (ninety years and upward), albeit it is one of those countries where, on the whole, old people (sixty years and upward) are relatively few. France takes the lead with respect to old people generally, but it has the small proportion 0.04 of the very old. Germany and Spain show the smallest number (0.03 and 0.02) of the very old people. Austria Hungary is something better. There are no statistical data of sufficient accuracy for the Russian Empire, but M. Leroy-Beaulieu is of opinion that the number of persons in Russia who are sixty years old and upward is abont forty-five in every thousand. Omitting Russia, Turkey, and some other small States, there are about eighteen million persons in Europe of the age of sixty and upward.

